# Activation of whole body by high levels of polyamine intake in rats

**DOI:** 10.1007/s00726-021-03079-4

**Published:** 2021-10-15

**Authors:** Takumi Teratani, Naoya Kasahara, Tetsuo Ijichi, Yasuhiro Fujimoto, Yasunaru Sakuma, Naohiro Sata, Joji Kitayama

**Affiliations:** 1 Division of Translational Research, Jichi Medical University, 3311-1, Yakushiji, Shimotsukeshi, Tochigi 329-0498 Japan; 2 Department of Surgery, Jichi Medical University, 3311-1, Yakushiji, Shimotsukeshi, Tochigi 329-0498 Japan; 3 Department of Surgery, Hyogo College of Medicine, 1-3-6 Minatojima, Chuo-ku, Kobeshi, Hyogo 663-8501 Japan

**Keywords:** Polyamine, Oral intake, Anti-aging, Transgenic rat, Living imaging

## Abstract

Polyamines are important to the survival and activation of organs and tissues via a homeostatic cell-metabolic process, and the polyamine content in cytoplasm decreases with aging. Decreases in cellular polyamine have been known to augment mutagenesis and cell death. Thus, supplementary polyamine in food is important to the prevention of aging. Here we show the anti-aging effects of oral intake of polyamine using luciferase-transgenic rats. Healthy rats, 10–12 weeks old, were given foods containing 0.01% and 0.1% (w/w) of polyamine, as compared a control food without polyamine, for 4 weeks. Using a bioimaging system, the photon intensities seen in the whole bodies and livers of rats consuming 0.1% of polyamine in food were stronger than those in rats consuming 0.01% and 0% of polyamine. However, there were no differences between groups in other characteristics, such as liver damage and body weight. In conclusion, we found that polyamine intake can activate cells throughout the whole body, providing an anti-aging effect.

**Supplementary Information:**

The online version contains supplementary material available at 10.1007/s00726-021-03079-4.

## Introduction

Polyamines are polycationic biogenic amines required for both eukaryotic cell growth and differentiation. They have attracted interest because of their multiple functions in cell biology (Mattoo et al. 2010; Tavladoraki et al. 2011), and studies have indicated that extracellular sources of polyamines are also important in metabolic processes (Rigueira et al. 2011). Polyamine synthesis decreases with aging, and a growing child produces the highest amounts of polyamine. Initially, polyamine is consumed in mother’s milk in early childhood. At least in animal models, the polyamine content in the mother’s milk, in combination with the immature de novo polyamine synthesis capacity in neonatal cells, influences the maturation and function of the neonatal intestinal mucosa and probably aids various immunological functions (Duchén and Thorell 1999). The polyamine (putrescine, spermine, and spermidine) concentrations in mother's milk and infant formulas were estimated by high-pressure liquid chromatography (HPLC; Romain et al. 1992). However, polyamines are naturally present at low concentrations in living organisms and in foods; thereby, the amount synthesized in situ may not be enough to meet health maintenance requirements. Therefore, supplementing polyamine is necessary for sufficient metabolic synthesis.

Meanwhile, aging is defined as a universal, progressive, and deleterious process occurring in cells and tissues, affecting most the living organisms. It is usually associated with progressive loss in functions across multiple systems, including sensation, cognition, memory, and motor control. Cognitive decline is a universal aspect of the aging process (Singh et al. 2011). In the aging process, as well as in most degenerative diseases, oxidant byproducts of the cellular metabolism lead to oxidative stress (Bianchi et al. 2007). Oxidative stress damages DNA in the host genome and induces apoptosis (Okita et al. 2007; Saeki et al. 2011). Since they are positively charged, spermine and spermidine interact with and stabilize DNA and RNA. Inhibition of these interactions between polyamine and DNA and/or low levels of spermine have been found to cause radio sensitivity, augmented mutagenesis, and cell death (Beckman and Ames 1997; Bianchi et al. 2007). Thus, as the amount of polyamine in the body increases, DNA and/or RNA damage is suppressed, which may greatly contribute to anti-aging effects.

Visualizing the state of cells across the whole body is very useful. Adenosine tri-phosphate (ATP) is an essential energy factor for living cells and vital activity, and is used in DNA and RNA synthesis. Like polyamine’s, ATP’s synthesizing activity decreases with aging (Haynes et al. 2010). We previously described the use of a luciferase transgenic (Luc-Tg) rat system, together with optical imaging techniques, to better understand cell and/or tissue conditions (Hakamata et al. 2006; Teratani and Kobayashi 2012). We previously described the use of a luciferase-based cell-viability assay that detects intracellular ATP levels in viable cells, which we used to assess the viability of Luc-Tg rat-derived islets and kidney, heart, small intestine, and liver samples (Doi et al. 2014; Iwai et al. 2012, 2014; Kasahara et al. 2013; Maeda et al. 2013; Negishi et al. 2011; Teratani and Kobayashi 2012).

In this study, we examined whether the long-term intake of polyamine-containing foods can increase whole body photon intensity levels in Luc-Tg rats. Additionally, we evaluated the safety of oral ingestion of polyamine at different concentrations, by examining major liver and kidney injury markers.

## Materials and methods

### Experimental animals

All experiments in this study were performed in accordance with protocols of the Jichi Medical School Guide for Laboratory Animals. The firefly Luciferase-expression transgenic Lewis rat (Luc-Tg LEW rat) was established in our laboratory as described previously (Hakamata et al. 2006; Teratani and Kobayashi 2011). Male Luc-Tg LEW rats weighing between 260 and 310 g, 10 and 12 weeks old, were used for the experiment. The animals were housed in a temperature- and humidity-controlled environment with a 12-h light/dark cycle and were provided with standard laboratory chow and water ad libitum.

### Polyamine-containing food

Test foods for the animal experiments were prepared by eliminating polyamine-rich materials from a standard rodent diet (0%; Oriental BIO Co., Ibaraki, Japan). For the test food with a high polyamine concentration (0.1%), synthetic spermine and spermidine (Wako Pure Chemical Industries, Ltd., Osaka, Japan) were mixed in doses of 80 µmol/kg and 360 µmol/kg, respectively, into the test food with a non-polyamine concentration. For the test food with a low polyamine concentration (0.01%), spermine and spermidine were mixed in doses of 8 µmol/kg and 36 µmol/kg, respectively, into the test food with a non-polyamine concentration. The test foods were prepared as pellets.

### In vivo bioluminescence imaging

In vivo luciferase imaging was performed using the in vivo imagining system (IVIS) and the IVIS Living Image software (Xenogen, Alameda, CA, USA). To detect photons from Luc-Tg rats’ whole bodies and tissues, D-luciferin (Promega, Madison, WI, USA) was injected into the tail veins of anesthetized rats (150 mg/kg; Negishi et al. 2011). Signal intensity was quantified as photon flux in units of photons/s/cm^2^/steradian in the region of interest.

### Measurement of polyamines

Amino acids were removed by cellulose phosphate column chromatography before the polyamine analysis because the polyamine levels in plasma were very low. We added 0.2 ml of 50% trichloroacetic acid to 1.8 ml plasma and centrifuged it for 10 min at 12,000 g. The supernatant thus obtained was neutralized with 6 N KOH and applied to a cellulose phosphate column (1 ml) previously equilibrated with a buffer containing 0.1 M boric acid–Na2CO3 and 0.025 M NaCl (pH 8.0). Amino acids were eluted with 10 ml of the same buffer and polyamines were then eluted with 3 ml of a buffer containing 0.2 M boric acid–Na2CO3 and 0.8 M NaCl (pH 8.0). The polyamine content was measured by HPLC as described previously (Igarashi et al. 1986; Sakata et al. 2003).

### Assessment of liver functions

Blood samples were obtained from each rat and centrifuged for 10 min at 3000 rpm, and serum was collected. Concentrations of markers of liver injury were analyzed using a FUJIFILM DRI-CHEM 3500 machine (FujiFilm, Tokyo, Japan) and FUJI DRY CHEM SLIDES (FujiFilm), respectively, for GOT/aspartate aminotransferase–PIII and GPT/alanine transaminase–PIII.

### Immunohistochemistry

An immunohistochemical study was performed on 10% formalin-fixed, paraffin-embedded tissues derived from each resected rat liver. Serial sections from each representative tissue block were deparaffinized and dehydrated. Tissue sections were then incubated with a primary monoclonal antibody against Ki67 (clone MIB-1, dilution 1:250; Dako, Tokyo, Japan), and incubated for 24 h in a cold room. The rhodamine- (dilution 1:1000) or FITC- (dilution 1:1000) conjugated secondary antibodies were applied for 30 min at room temperature. Eight-micron paraffine-embedded sections were stained with hematoxylin and eosin for conventional morphological evaluation. Paraffin sections of the post-operation days 7 (POD7) rat livers were prepared.

### Statistical analysis

Data are presented as means ± standard deviations. The statistical analysis was conducted using a Student’s *t*-test and a non-repeated measurement analysis of variance, followed by a Bonferroni post hoc comparison test. We considered *P* values < 0.05 to indicate statistical significance.

## Results

### Polyamine-containing foods

**Fig. 1:**
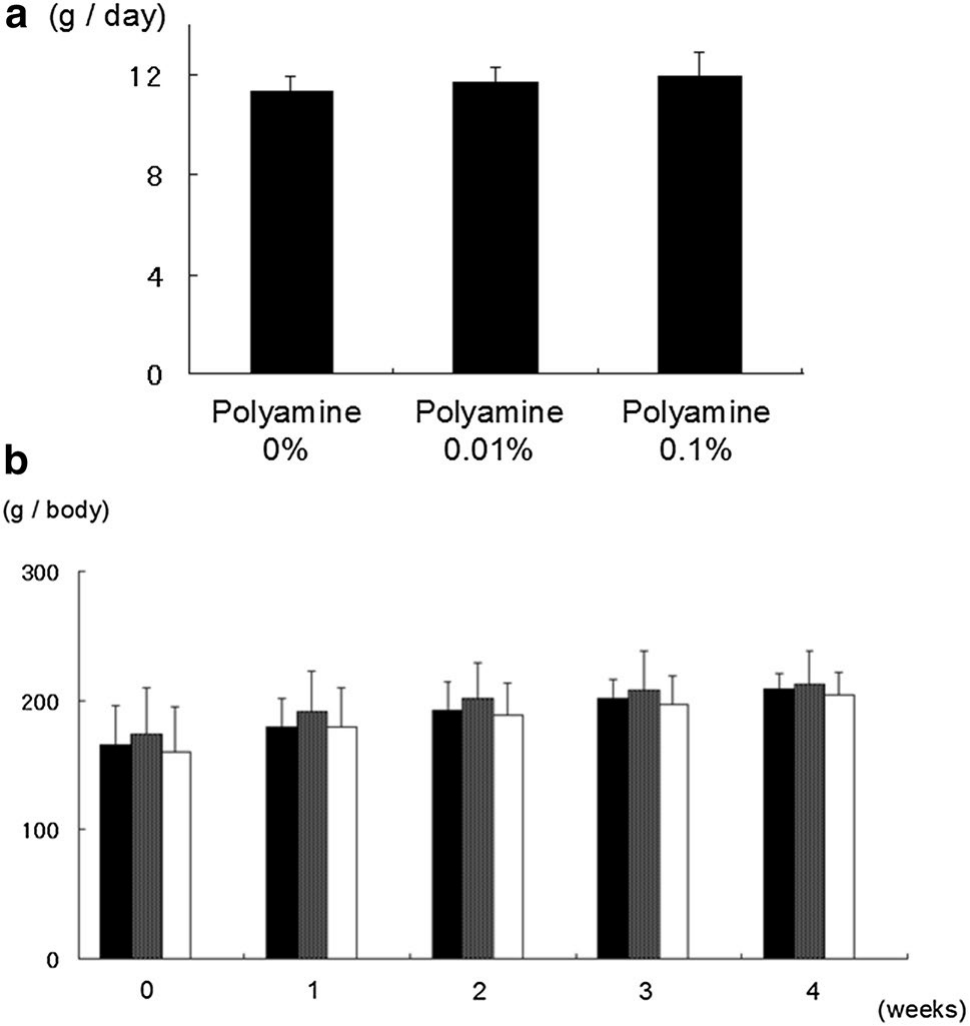
Analysis of the conditions of rats consuming the test foods (*n* = 6 each). **a** Daily intake of 10-week-old Luc-Tg rats. There was no change in intake amounts due to changes in polyamine content. **b** Furthermore, there was no weight change. The black bar indicates 0% of polyamine in food, the gray bar indicates 0.01% of polyamine, and the white bar indicates 0.1% of polyamine

We tested 3 different polyamine-containing foods. Neither the amounts of food consumed (Fig. 1a) nor the body weights (Fig. 1b) differed between groups. Thus, these results suggest that polyamine supplementation does not induce asitia and/or growth failure in rats. In this experiment, we used these foods to test the anti-aging effects of polyamine in Luc-Tg rats.

### Effects of polyamine-containing foods on photon intensity

The new field of in vivo imaging is being developed with luminescent biotechnology, and involves the real-time visualization of complex cellular processes in living animals. In inbred Tg rats with firefly luciferase, we compared levels of photon intensity between a young Luc-Tg rat (6 weeks old) and an older Luc-Tg rat (28 weeks old) using an in vivo imaging system (Online Resource 1). The photon intensity across the whole body was stronger in the young Luc-Tg rat than in the older Luc-Tg rat. Additionally, the photon intensity levels of organs and tissues decreased with age (data not shown).

**Fig. 2:**
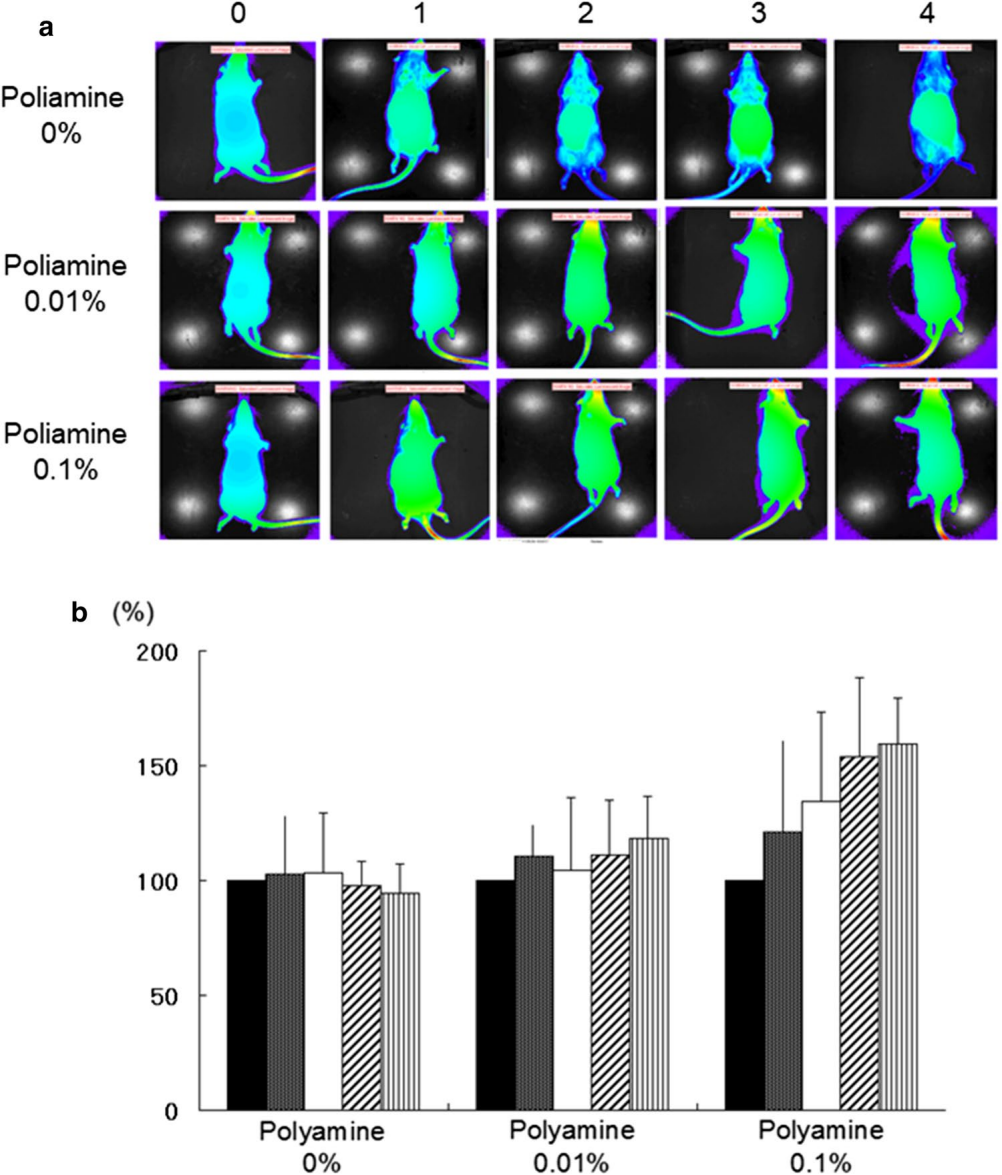
Changes in photon intensity based on intake of polyamine (*n* = 6 each). **a** Kinetics of photon intensity levels over several weeks. **b** The value of the photon intensity at the 0th week was set to 100%. **c** In the bar graph, each groups’ values are shown at 0, 1, 2, 3, and 4 weeks from left to right. The food with 0.01% polyamine has the same polyamine content as normal commercial foods

Next, we investigated the anti-aging effects of test foods supplemented with polyamine in Luc-Tg rats. The photon intensity levels increased with higher concentrations of polyamine, and did not change in the group without supplementation (Fig. 2a). Furthermore, time-dependent changes in photon intensity levels were quantified from images taken with the IVIS Living Image software (Fig. 2b). When comparing intensity levels after 4 weeks, the photon intensity had increased by 1.2-fold in the group consuming 0.01% of polyamine in food (118.607 ± 17.911%) and by 1.6-fold in the group consuming 0.1% of polyamine (159.543 ± 19.897%), compared with the group consuming 0% of polyamine (94.505 ± 12.579%). Thus, these results suggest systemically anti-aging effects of polyamine consumption in rats. Additionally, the anti-aging effect was found to change in proportion to the polyamine concentration.

### Effects of polyamine on activation of the liver

**Fig. 3:**
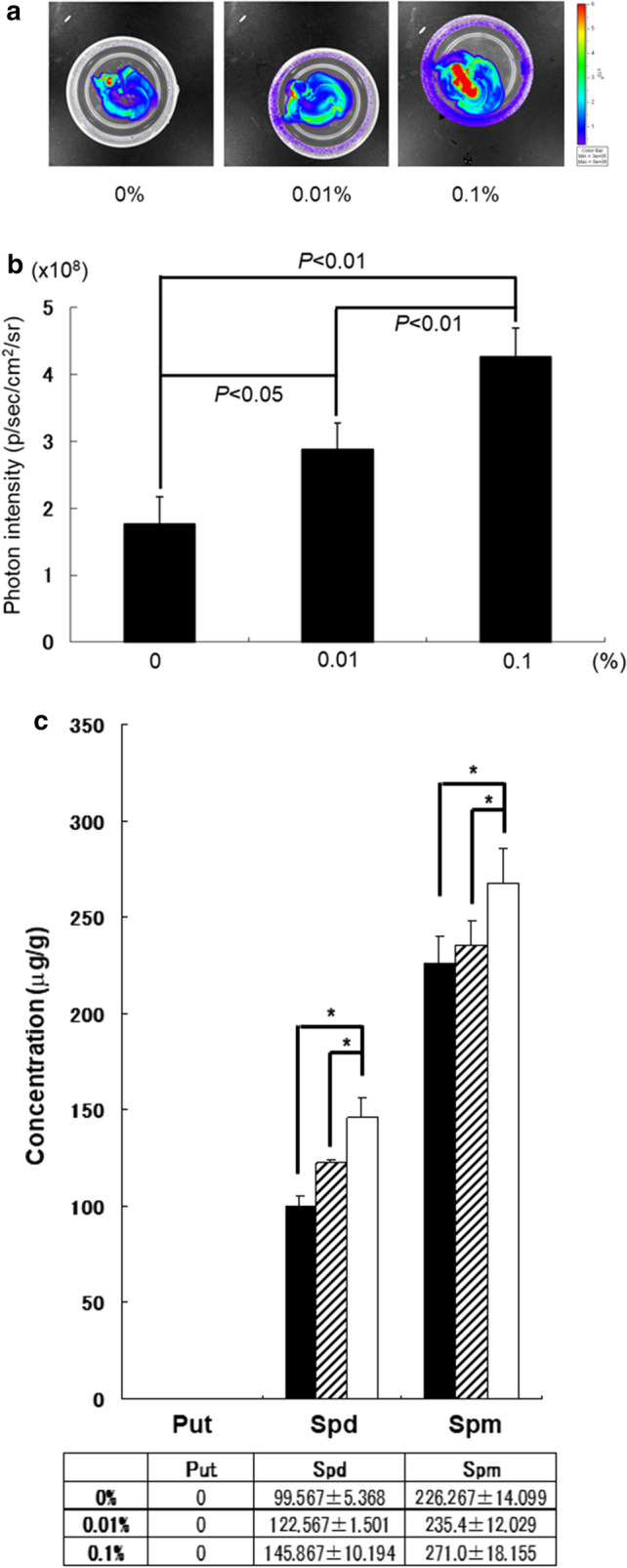
Photon images of rat livers based on polyamine intake (*n* = 6 each). **a** After 4 weeks of oral intake, the rats’ whole livers were removed using by IVIS. **b** Photon intensity values are shown for rats consuming 0%, 0.01%, and 0.1% of polyamine in food. Photon levels of rats consuming foods with 0% and 0.01% of polyamine were significantly lower than those of rats consuming 0.1% of polyamine in food (*P* < 0.01). **c** The polyamine concentrations of rat livers were examined in each experimental group using high-pressure liquid chromatography. The black bar indicates 0% of polyamine in food, the gray bar indicates 0.01% of polyamine, and the white bar indicates 0.1% of polyamine (*P* < 0.01)

We analyzed the impacts of polyamine intake for 4 weeks on the livers of Luc-Tg rats using IVIS (Fig. 3a). The photon intensity level increased with consumption of polyamine, similar to results from our analysis of the whole body. Next, the photon intensity levels in liver were quantified from these IVIS image files based on the test foods (Fig. 3b). The photon intensity level increases in the liver were proportional to the polyamine concentration increases, with the same levels in rats consuming 0.1% of polyamine and in young rats at 6 weeks of age (Online Resource 2). We investigated the accumulation of polyamines in rat livers by HPLC analysis (Fig. 3c). Rats consuming 0% and 0.01% of polyamine had significant differences in liver polyamine concentrations; however, those consuming 0.1% of polyamine had a significant difference. Thus, these results suggest that polyamine has a direct effect on anti-aging and/or cell viability of several organs.

### Analysis of liver toxicity by test food group

**Fig. 4:**
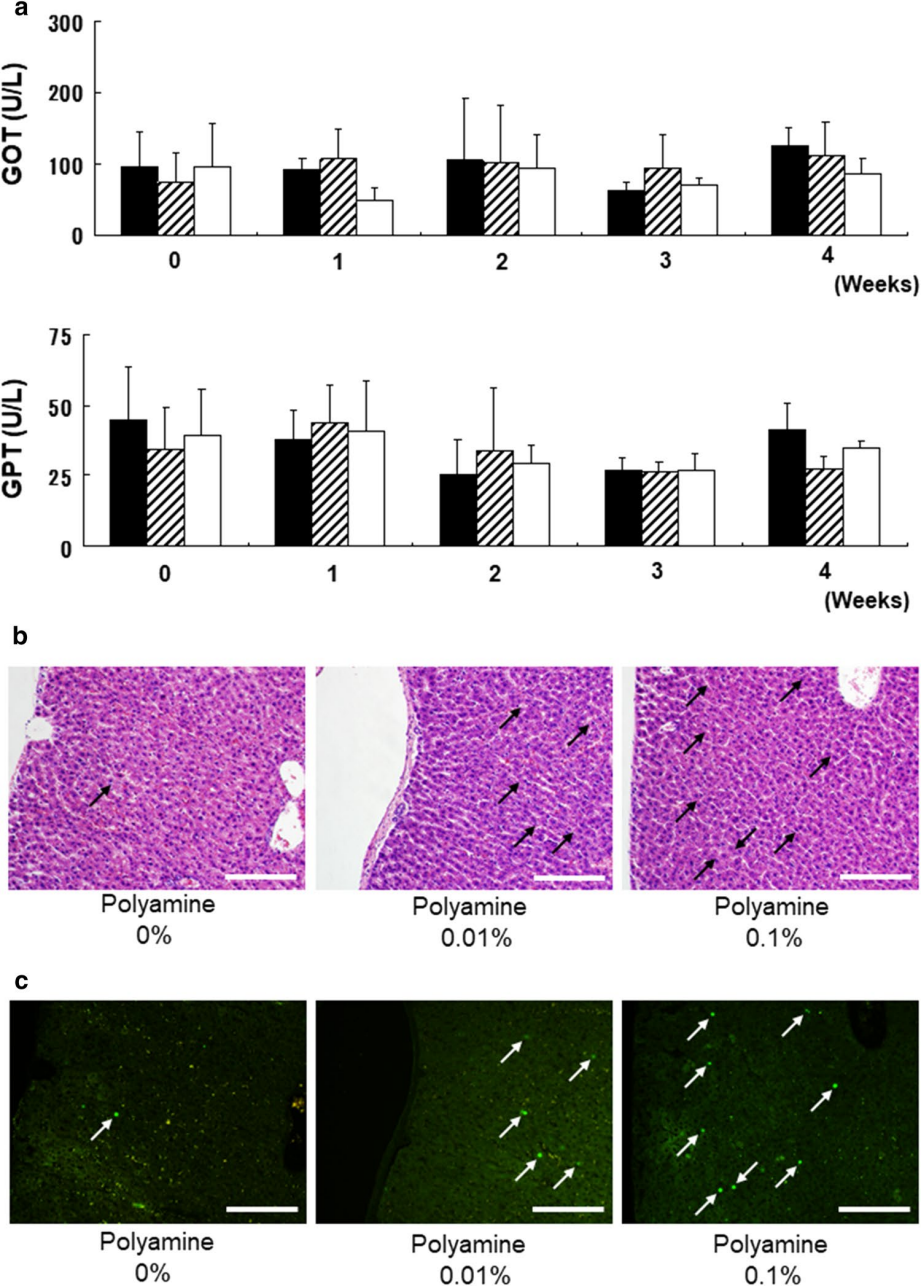
Analysis of serum and pathological specimens. **a** Serum levels of aspartate aminotransferase and alanine transaminase are shown at 0–4 weeks of oral intake of foods with polyamine (*n* = 6 each). The black bar indicates 0% of polyamine in food, the gray bar indicates 0.01% of polyamine, and the white bar indicates 0.1% of polyamine. Representative **b** hematoxylin and eosin–stained and **c** Ki-67-stained sections of liver. Arrows indicate Ki-67-positive cells of hepatocytes. Original magnification × 100. Scale bars, 200 µm

Finally, we investigated the polyamine toxicity within groups by testing liver sections and serum in rats. Biochemical parameters such as GOT and GPT were not increased in mice consuming polyamine-containing foods, compared to those consuming food without polyamine (Fig. 4a). Furthermore, there were no pathological differences in liver sections between groups (Fig. 4b). However, Ki67-positive cells of cell proliferation maker were increased in rats consuming polyamine-containing test foods (0.01% and 0.1% polyamine), compared with controls (Fig. 4c). However, TdT-mediated dUTP nickend labeling (TUNEL)-positive cells of an apoptosis marker were not found to significantly differ across groups (data not shown). Thus, 4 weeks of consuming 0.1% of polyamine does not lead to toxicity. Furthermore, the function of polyamine was clear based on the inhibition of apoptosis and on liver proliferation.

## Discussion

The polyamines spermine, spermidine, and putrescine are ubiquitous cell components that are essential for cell proliferation and differentiation (Pegg 1988). They attract interest because of their multiple functions in cell biology, including cell cycle regulation, gene expression, and signal transduction, among many others (Agostinelli et al. 2010; Bachrach et al. 2001; Childs et al. 2003). Polyamine levels decrease with age in many organisms (Scalabrino and Ferioli 1984), although decreases are polyamine and tissue specific. For instance, Nishimura et al. (2006) measured polyamine levels in 14 different tissues in 3-, 10-, and 26-week-old female mice and found that spermidine levels decreased in 11 out of the 14 tissues. In contrast, spermine decreased only in the skin, heart, and muscles. Putrescine levels were very low in all tissues at all ages. Eisenberg et al. (2009) followed the consequences of external spermidine administration in various model organisms, including yeast, worms, flies, mice, and human cells (Eisenberg et al. 2009; Minois et al. 2011). Spermidine increased the chronological life span in wild-type yeast, as well as the remaining replicative life span in old yeast cells. In contrast, a Δspe1 yeast mutant unable to synthesize polyamines was short-lived. This decreased life span was rescued by the addition of spermidine, as well as of putrescine. Additionally, spermidine supplementation increased the life span of the nematode worm *Caenorhabditis elegans* by 15% and of the fly *Drosophila melanogaster* by up to 30%. At the cellular level, spermidine increased survival of human peripheral blood mononuclear cells after 2 days from 15% in controls to 50% by preventing death from necrosis.

The main source of exogenous polyamines is dietary (Larqué et al. 2007), and the intestinal lumen is the main exogenous source for the body (Hosomi et al. 1987; Osborne and Seidel 1990). Since the level of polyamines decreases with age in animal organs (brain, kidney, spleen, and pancreas), it has been suggested that maintenance of the polyamine level from the diet is important to the functioning of various organs in the elderly (Das and Kanungo 1982; Larqué et al. 2007). A high intake of spermine is associated with a decreased risk of food allergies among suckling rats, as well as human children, due to the contribution of spermine to maturation of both the immune system and the small intestinal mucosa (Ali et al. 2011; Dandrifosse et al. 2000; Dufour et al. 1988; Löser 2000).

At the same time, aging is a fundamental biological phenomenon of great medical importance (Anand et al. 2012). It is well known that many age-related behavioral changes occur even in the absence of specific, age-related, neurodegenerative diseases, such as Alzheimer's disease or Parkinson's disease (Butterfield et al. 1999). D-galactose causes age-related changes in different animal models. It has been reported that free radicals are increased in animals receiving d-galactose, and the free radical theory of aging shows that these increased free radicals might contribute to the mechanisms underlying age-related degenerative diseases (Ho et al. 2003). Oxidative stress is the result of an imbalance between generation and scavenging of reactive oxygen species (ROS). Old cells have higher levels of ROS than young cells (Hagen et al. 1997; Lee et al. 1999). Senescent states of cells can be readily induced by sub-lethal doses of pro-oxidants (Chen and Ames 1994; Chen et al. 1998). Thus, any adjustment to the ROS balance is expected to prevent or restore cellular senescence (Kim and Wu 2009; Kim and Park 2012). Endogenous free radical scavengers such as polyamines can inhibit the action of ROS. Furthermore, polyamines have been shown to indirectly increase HO-1 content and antioxidant protection (von Deutsch et al. 2005). Furthermore, ATP synthesis ability decreases in major organs such as the liver and brain with aging (Alemany et al 1988; Eckert et al. 2013). The reason is that the quality of mitochondria deteriorates due to aging, and the increase in oxidative stress reduces the amount of ATP produced (Rodríguez et al. 2008).

Recently, we found that perioperative oral polyamine administration attenuates liver ischemia–reperfusion injury and promotes liver regeneration (Doi et al. 2019; Okumura et al. 2016). In a liver ischemia–reperfusion injury, the number of PCNA (Proliferation cell nuclear antigen)-positive and Ki67-positive cells were increased in the polyamine group compared to the non-polyamine group. Okumura et al.’s (2016) report suggests that postoperative cell proliferation, DNA synthesis, and liver regeneration were promoted by polyamine treatment. HE staining indicates that there is no abnormality in the liver pathologically, and at the same time, which cells are Ki67-positive. It has also been found that the Ki67-positive rate decreases with aging (Kalaz et al. 2014). This figure shows that oral intake of polyamines in aged rats restores Ki67-positive cells to the same level as in young rats. Furthermore, in a study investigating the effects of polyamines, our team found that oral polyamines have a wide range of therapeutic effects in vitro and in vivo, including promotion of anti-aging, testicular activation, cartilage regeneration, and short bowel syndrome (Preparation of manuscripts). Especially, based on short bowel syndrome’s basic research data, we took the lead in conducting a clinical trial of oral polyamines in a patient with short bowel syndrome after obtaining approval from the Ethics Committee in the Jichi Medical University Hospital (Approval No. 16-47). As a result, a significant improvement in quality of life was confirmed, including an improvement in nutritional status and a significant decrease in the frequency of diarrhea. Furthermore, oral polyamine had no adverse effects on other medical conditions (Preparation of manuscript).

In conclusion, using an in vivo imaging system we found here that intake of polyamine activates cells in the whole body and provides an anti-aging effect.

## Supplementary Information

Below is the link to the electronic supplementary material.

Online Resource 1 Photon imaging of a young rat (6 weeks old) and an aged rat (28 weeks old). The photon intensity value of the young rat was higher than that of the older rat

Online Resource 2 Photon imaging of a young rat’s liver (6 weeks old) and an aged rat’s liver (28 weeks old). The photon intensity value of the young rat was higher than that of the older rat

## Data Availability

Only if you make a prior notice to the corresponding author, all of the data is fully available without restriction.
