# Supplementary figures and images for: Activation of whole body by high levels of polyamine intake in rats
Source: Amino Acids. 2021 Oct 15;53(11):1695–703. doi: 10.1007/s00726-021-03079-4 (PMC8592999; doi:10.1007/s00726-021-03079-4)

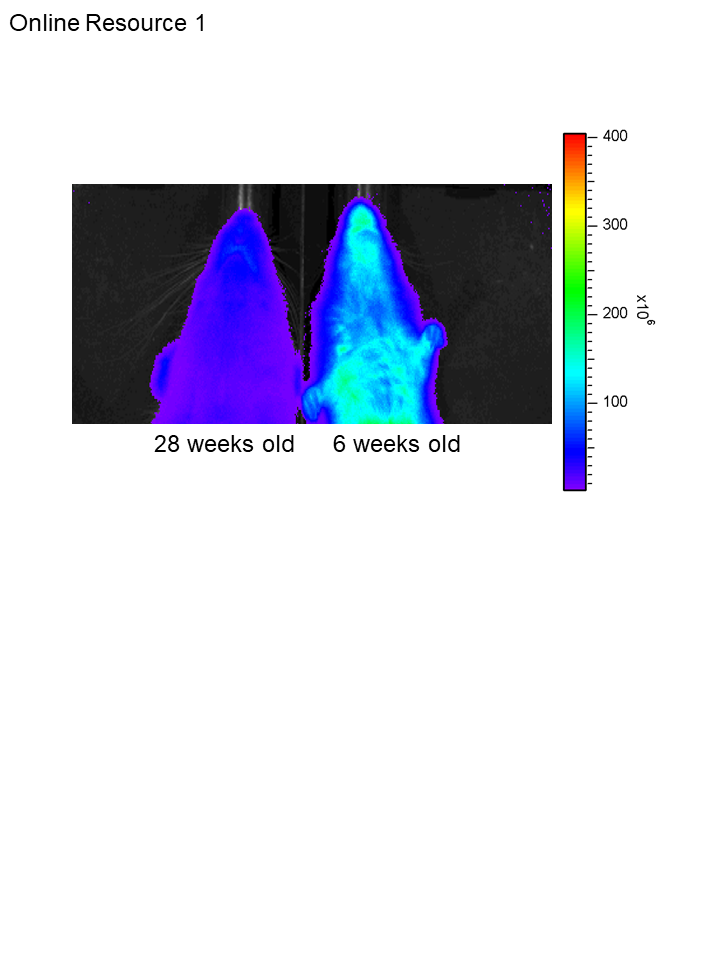

Supplement: Supplementary file 1 — Online Resource 1 Photon imaging of a young rat (6 weeks old) and an aged rat (28 weeks old). The photon intensity value of the young rat was higher than that of the older rat [file 726_2021_3079_MOESM1_ESM.tif]

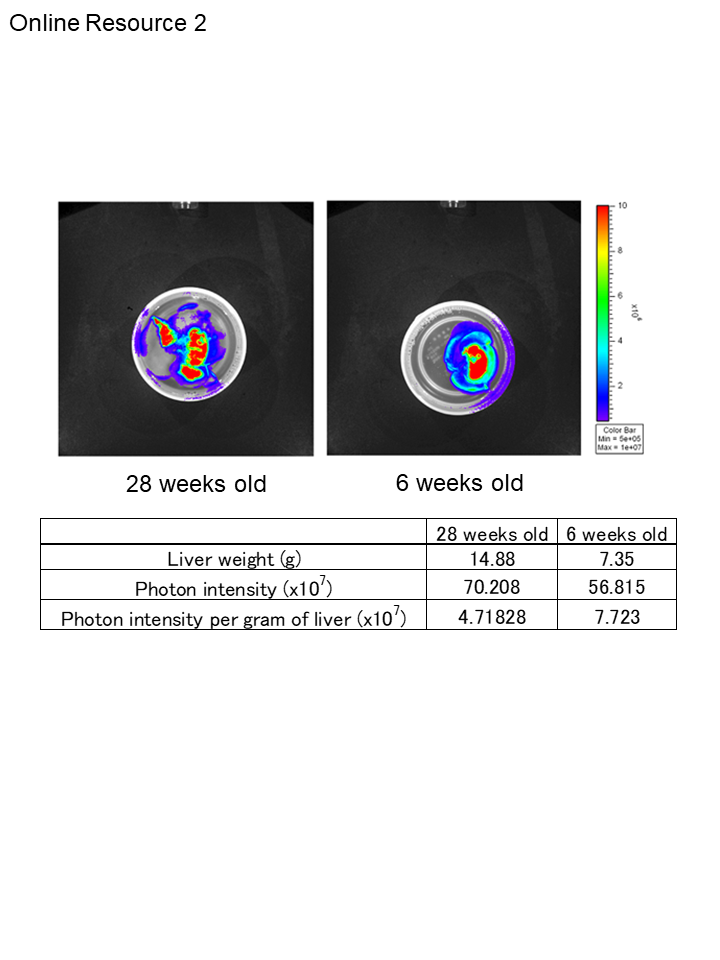

Supplement: Supplementary file 2 — Online Resource 2 Photon imaging of a young rat’s liver (6 weeks old) and an aged rat’s liver (28 weeks old). The photon intensity value of the young rat was higher than that of the older rat [file 726_2021_3079_MOESM2_ESM.tif]
